# Immunopurification of adenomatous polyposis coli (APC) proteins

**DOI:** 10.1186/1756-0500-6-429

**Published:** 2013-10-25

**Authors:** Kerryn L Elliott, Bruno Catimel, Nicole L Church, Janine L Coates, Antony W Burgess, Meredith J Layton, Maree C Faux

**Affiliations:** 1 Ludwig Institute for Cancer Research-Parkville branch, Parkville, VIC 3050, Australia; 2 The University of Melbourne, Parkville, VIC 3050, Australia; 3 Current address: The University of Gothenburg, Medicinaregatan 9C 41390, Gothenburg, Sweden; 4 Current address: Ludwig Institute for Cancer Research-Austin Branch, Austin Hospital, Level 5, Olivia Newton-John Cancer and Wellness Centre, Studley Road, Heidelberg, VIC 3084, Australia; 5 Current address: The Walter and Eliza Institute of Medical Research, 1G Royal Parade, Parkville, VIC 3052, Australia

**Keywords:** Adenomatous polyposis coli, Colon cancer, Antibody, Protein purification

## Abstract

**Background:**

The *adenomatous polyposis coli* (*APC*) tumour suppressor gene encodes a 2843 residue (310 kDa) protein. APC is a multifunctional protein involved in the regulation of β-catenin/Wnt signalling, cytoskeletal dynamics and cell adhesion. *APC* mutations occur in most colorectal cancers and typically result in truncation of the C-terminal half of the protein.

**Results:**

In order to investigate the biophysical properties of APC, we have generated a set of monoclonal antibodies which enable purification of recombinant forms of APC. Here we describe the characterisation of these anti-APC monoclonal antibodies (APC-NT) that specifically recognise endogenous APC both in solution and in fixed cells. Full-length APC(1–2843) and cancer-associated, truncated APC proteins, APC(1–1638) and APC(1–1311) were produced in Sf9 insect cells.

**Conclusions:**

Recombinant APC proteins were purified using a two-step affinity approach using our APC-NT antibodies. The purification of APC proteins provides the basis for detailed structure/function analyses of full-length, cancer-truncated and endogenous forms of the protein.

## Background

The *adenomatous polyposis coli (APC)* gene is mutated in the germline of individuals with familial adenomatous polyposis (FAP) and in more than 80% of sporadic colorectal cancers [1-3]. *APC* encodes a large, multifunctional protein (2843 aa, 310 kDa) and contains protein-binding domains that have implicated APC in a range of cellular functions. APC is best characterised for its role in the down-regulation of β-catenin and the Wnt signalling pathway through interactions with β-catenin and Axin [4-8]. APC also functions in cytoskeletal organisation, cell migration and adhesion [9,10] via interactions with cytoskeletal proteins, such as tubulin, actin, EB1 and discs large protein [11-14].

*APC* mutations are generally missense mutations that introduce a premature stop codon, leading to expression of truncated APC proteins [1]. The majority of mutations in *APC* are confined to a mutation cluster region (MCR) [2] encompassing the β-catenin and Axin binding sites. Truncation of APC disrupts key binding sites in the C-terminus of the protein, including interactions with the Wnt signalling proteins and both actin and microtubule cytoskeletons. It is now established that APC truncation leads to aberrant regulation of β-catenin which results in increased transcription of Wnt target genes [15,16].

Despite the importance of APC in colorectal cancer, little is known about the biophysical properties and/or structure of the APC protein or its cancer-truncated forms. Limited structural information on APC has come from studies using small fragments of APC, usually in complex with other proteins. The N-terminus of APC was crystalised as a coiled-coil dimer [17,18], and the 15 and 20 aa repeats were crystalised as fragments with β-catenin [19,20]. However, as these studies used small fragments rather than the full-length APC protein, the structural differences and implications for protein binding between full-length or the truncated APC are not yet known.

In the present study, we describe the characterisation of new APC monoclonal antibodies and their use in the purification of recombinant forms of APC. APC monoclonal antibodies were generated to the N-terminus of APC, and antibody clones were selected by a combination of ELISA, immunoprecipitation and biosensor analysis. The antibodies were then further characterised by immunoprecipitation and immunofluorescence of endogenous APC. Full length (fl-APC) and truncated APC proteins (APC(1–1638) and APC(1–1311)) were generated using baculoviral-mediated expression in Sf9 cells and purified using a two-step affinity strategy involving immobilised metal affinity chromatography (IMAC) and APC monoclonal antibodies.

## Results

### Generation of novel APC monoclonal antibodies

The N-terminus of APC has been shown to form a coiled-coil structure and dimerise in solution [21]. An N-terminal fragment of APC (residues 1–61) was used as an antigen to raise monoclonal antibodies. This region of APC forms a dimer (not shown) and was therefore thought to mimic the structure of the same region in full-length APC.

**Figure 1:**
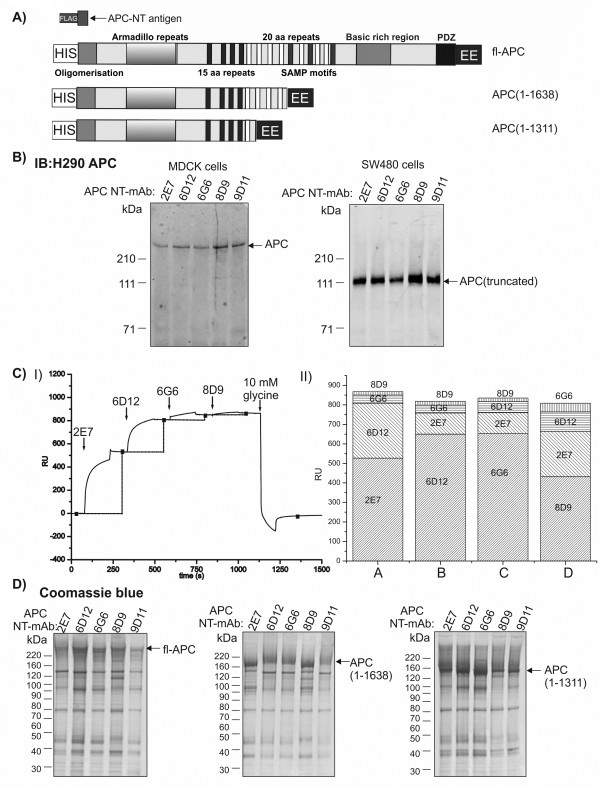
**APC-NT mAbs recognize endogenous and recombinant full-length and truncated APC proteins in solution. A)** Schematic diagram of structures of recombinant APC proteins. The APC-NT antigen (APC residues 1–61 with an N-terminal FLAG-tag) was expressed and purified from *E.coli* and used to generate the APC-NT mAbs. Full-length (fl-APC) and truncated recombinant APCs APC(1–1638) and APC(1–1311) were expressed in Sf9 cells with N-terminal HIS and C-terminal EE tags. The protein domains of APC are indicated: Oligomerisation, Armadillo repeats, 15 aa repeats, 20 aa repeats, SAMP motifs, basic rich domain, PDZ domain. Note: the EE-epitope tag was not used for the final affinity purification. **B)** Immunoprecipitation of endogenous full-length and truncated APC. APC-NT mAb immunoprecipitates from MDCK cells (1 mg protein, left panel) and SW480 colorectal carcinoma cells (1 mg protein, right panel) were immunoblotted (IB) with anti-APC H290. **C)** Biosensor analysis shows overlapping and non-overlapping epitopes for the APC-NT mAbs. I) Representative sensorgram showing sequential injection of APC-NT mAbs and binding to APC-NT antigen. II) Stack graph showing increases in binding (RU) to APC-NT antigen upon sequential injections with different APC-NT mAb clones. Four combinations of APC-NT mAb clones injected sequentially are represented by shaded areas **(A-D)**. Column A corresponds to the sensorgram in I). 2E7 and 8D9 have different epitopes whereas 6D12 and 6G6 share overlapping epitopes. **D)** Immunoprecipitation of recombinant APC proteins expressed in Sf9 cells. APC-NT mAb (2 μg) immunoprecipitates from Sf9 cell lysates (2×10^7^ cells) expressing fl-APC (left), APC(1–1638) (middle) or APC(1–1311) (right) were resolved by SDS-PAGE and immunoprecipitated proteins were visualised with Coomassie blue. Note protein immunoprecipitated by 6D12 and 6G6 migrate slightly differently. The relative positions of the molecular markers are indicated.

Anti-APC-NT mouse monoclonal antibodies were produced and clones were screened for immunoreactivity to the immunizing antigen by ELISA and surface plasmon resonance (BIAcore) (not shown). Clones that recognised APC-NT were isotyped and analysed for their ability to immunoprecipitate endogenous APC from MDCK epithelial cells (containing wild-type APC) and SW480 colorectal carcinoma cells (containing mutated, truncated APC [22]) (Figure 1B). Both full-length (wild-type) and truncated APC were immunoprecipitated by APC mAb clones 2E7, 6D12, 6G6, 9G11 (all IgG) and 8D9 (IgG2a) (Figure 1B). Therefore APC-NT antibodies can be used to purify endogenous APC proteins, both wild-type and cancer mutated, truncated APC.

In contrast, APC-NT mAbs did not detect APC proteins by Western blot analysis. Cell lysates from MDCK or SW480 cells were separated by SDS-PAGE and immunoblotted with APC-NT or commercial APC antibodies. Only the commercial antibody detected wild-type or truncated APC proteins on immunoblots (not shown). This suggests that APC-NT mAbs can recognise both full-length and truncated endogenous APC in solution, supporting the prediction that the antigen used had a similar structure to the same region in endogenous, native APC. APC-NT mAbs are unlikely to recognise a linear epitope, as the epitope is not retained upon denaturation of endogenous APC during SDS-PAGE and immunoblotting. Recognition of a conformation-dependent epitope is likely to contribute to the specificity of the APC-NT mAbs for endogenous APC.

These APC-NT mAbs were analysed using BIAcore to determine whether their epitopes overlapped. Purified APC-NT was immobilised onto a BIAcore CM5 chip and the antibodies were injected sequentially over the immobilised peptide. Binding of mAb 6D12 inhibits binding of 6G6 and 8D9, and vice versa, suggesting they have at least partially overlapping epitopes, but mAb 2E7 appears to bind independently of the other three antibodies (Figure 1C), suggesting that at least 2 different epitopes on APC-NT are recognised by the APC mAbs.

### Generation of APC-encoding baculoviruses

Baculoviruses encoding fl-APC and truncated APC proteins (APC(1–1638) and APC(1–1311)) containing N-terminal HIS and C-terminal EE [23] affinity tags were generated using the Bac-N-Blue system (Figure 1A). Recombinant APC-encoding baculoviruses were plaque-purified and used for expression of recombinant APC in Sf9 cells. Cells were infected at an MOI of 5 (fl-APC) or an MOI of 1 (truncated APCs) for 72 hr which was found to produce the highest levels of recombinant APC.

The EE-tag was inefficient for purification of the relatively low levels of recombinant APC expressed by the baculovirus. The APC-NT mAbs were therefore tested for their ability to immunoprecipitate recombinant APC proteins, fl-APC, APC(1–1638) or APC(1–1311) from APC baculoviral-infected Sf9 cells (Figure 1D). In APC-NT Ab immunoprecipitates, predominant bands were detected at the expected molecular weights of 310, 190 and 150 kDa, respectively for fl-APC, APC(1–1638) and APC(1–1311) (Figure 1D, left, middle and right panels, respectively). The APC(1–1638) immunoprecipitated using the APC mAbs 2E7 and 8D9 appeared to have a slightly altered mobility on SDS-PAGE compared to that immunoprecipitated with 6D12, 6G6 and 9D11. However, the concentrations of the antibodies in the immunoprecipitations may differ, thus altering the apparent mobility. Taken together this data shows that each of the 5 APC-NT mAb clones, selected for their ability to recognise endogenous APC in solution, also recognise recombinant full-length and truncated APC expressed in insect cells. All five APC-NT mAbs were used to generate an APC mAb affinity matrix by covalently cross-linking 5 mg of antibody (1 mg each of 2E7, 6D12, 6G6, 8D9 and 9G11) to 5 ml of Protein G-Sepharose beads.

### Characterisation of APC-NT mAbs by immunofluorescence and APC-directed siRNA treatment

**Figure 2:**
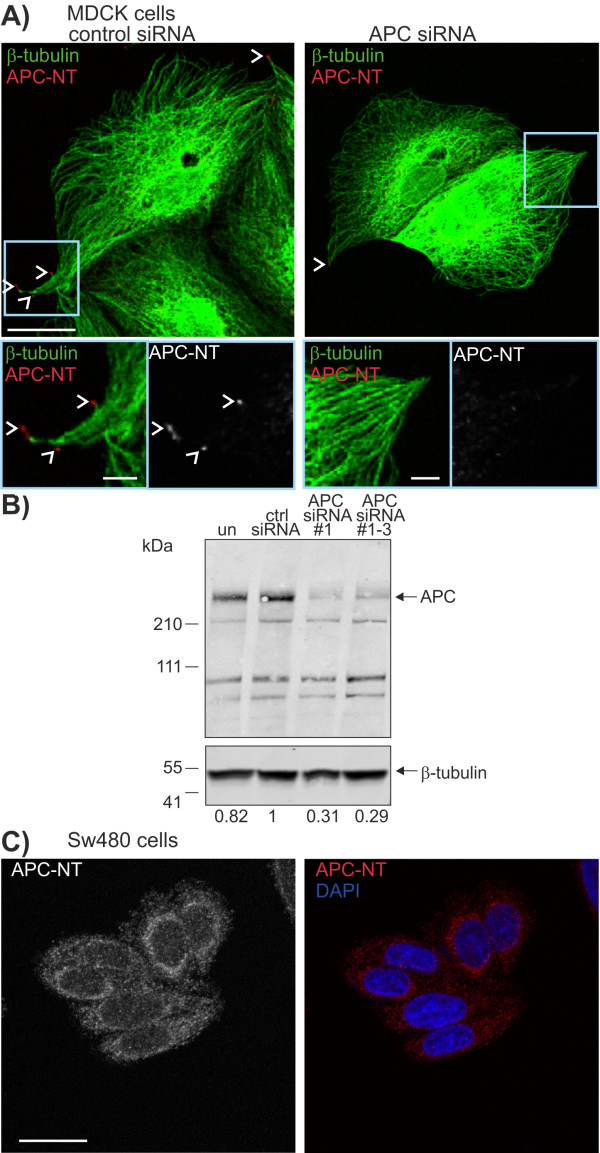
**APC-NT mAbs specifically detect APC at the ends of microtubules. A)** MDCK cells transfected with control siRNA (top) or APC siRNA (bottom) were immunostained for APC (APC-NT mAb, 6D12 (red)) and β-tubulin (green). Distinct clusters of APC at the ends of microtubules (arrowheads) are detected by the APC-NT mAb in control but not APC depleted cells, scale bar 20 μm. Enlarged view of insets is shown (right, merged image (left) and single channel grayscale for APC (right)). Shown are single section confocal images, scale bar 5 μm. **B)** Depletion of cellular APC by siRNA. APC levels following siRNA transfection were assessed by immunoblot analysis (with H290 Ab from Santa Cruz) in cell lysates. Tubulin was used as a loading control. Numbers below blot are the normalised ratio of APC protein compared to control siRNA. The relative positions of molecular weight markers are indicated. **C)** SW480 CRC cells were immunostained for APC (APC-NT) and costained with DAPI. APC staining is diffusely cytosolic and is not detected in clusters at the ends of microtubules. Shown are single channel grayscale image for APC (left) and merged image (APC, red and DAPI blue), scale bar 20 μm.

We next assessed whether the APC-NT mAbs recognised endogenous APC protein by immunofluorescence. APC-NT mAb staining in MDCK cells was analysed by confocal microscopy and compared to a commercially available APC antibody (anti-APC-H290). In subconfluent epithelial cells, APC decorates the ends of microtubules at cell protrusions [24,25]. The five APC-NT antibody clones each demonstrated the expected staining pattern with concentrated clusters at the ends of microtubule (Figure 2A, representative image shown for mAb 6D12). All APC antibodies recognize APC at microtubule protrusions, however only APC H290 demonstrated nuclear staining and none of the antibodies detected APC at the centrosomes.

We confirmed the specificity of APC-NT antibodies by comparing their immunofluorescent staining pattern in MDCK cells treated with control siRNA with those in which APC levels were depleted using APC-specific siRNAs. Transfection of MDCK cells with siRNAs targeting APC reduced the level of APC by 70% (Figure 2B). APC staining was still detectable in a proportion of cells, presumably reflecting residual APC protein. However, the typical staining of APC at the ends of microtubules was absent in the majority of APC siRNA transfected cells for each APC-NT mAb (Figure 2A, representative image for APC-NT 6D12). Thus each of the APC-NT mAbs specifically recognise endogenous APC localised in clusters at the ends of microtubules.

We next investigated the distribution of APC in SW480 CRC cells with the APC-NT antibodies (Figure 2C, representative image for APC-NT 6G6). In contrast to cells expressing full-length wild-type APC, the truncated APC protein in SW480 cells was not detected at the ends of microtubules but instead was diffusely cytosolic with some concentration of staining in the perinuclear region. This distribution is consistent with the loss of mictrotubule binding in truncated APC and concomitant alterations in cell migration that occur upon *APC* mutation.

### Purification of recombinant APC proteins

**Figure 3:**
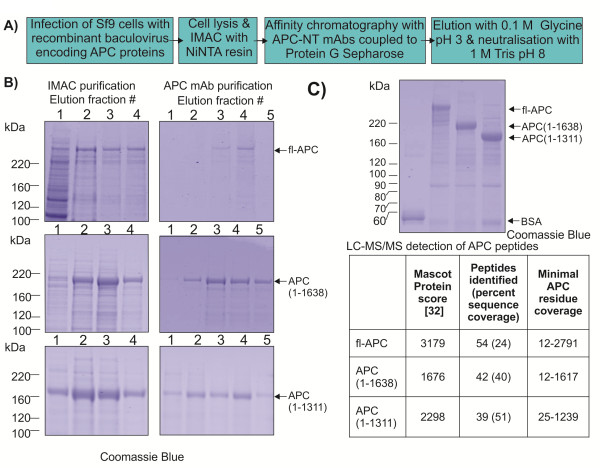
**Purification of recombinant APC proteins by a two-step method using IMAC and APC-NT mAb affinity chromatography. A)** Schematic diagram depicting the purification scheme used to purify recombinant APC proteins. **B)** Purification of recombinant APC proteins. Sf9 cells (2×10^9^) infected with either fl-APC, APC(1–1638) or APC(1–1311) were and purified using Ni-NTA resin. Proteins were eluted using a stepwise gradient of 20, 50, 100 and 250 mM imidazole. 1% of each fraction was resolved using 4-12% Bis-Tris SDS-PAGE and stained with Coomassie blue (Left panel, 1 = 20 mM, 2 = 50 mM, 3 = 100 mM, 4 = 250 mM). Pooled fractions containing APC were applied to a 5 ml APC-NT mAb column and eluted with 0.1 M Glycine pH 3 in 5× 2 ml fractions (right). 1% of each fraction was resolved as above. The expected sizes of APC proteins are indicated by arrows and relative positions of molecular weight markers in kDa are indicated. **C)** Purified recombinant APC proteins. APC proteins were purified using the optimized strategy **(A)**. The amount of APC was estimated by comparison to a band representing 1 μg BSA. The expected size of APC proteins are indicated by arrows and relative positions of molecular weight markers are indicated. Purified APC bands were excised and analysed using LC-MS/MS. MASCOT Protein score [26], peptide number, and amino acid coverage are indicated for each protein.

Having confirmed the specificity of the APC-NT mAbs, a two-step purification scheme for recombinant APC was developed (Figure 3A). Recombinant fl-APC, APC(1–1638) or APC(1–1311) was first purified from clarified Sf9 cell lysates by IMAC. Fractions from this step contained a predominant band at the expected molecular weight as well as a number of minor bands (Figure 3B, left panels). The fractions containing recombinant APC were purified further using APC-NT mAb affinity chromatography. To ensure maximal binding efficiency, the pooled fractions were re-circulated over the APC-NT mAb column three times. Recombinant APC was eluted by disrupting the APC: antibody interaction using low pH. Fractions from this step also contained a predominant band at the expected molecular weight and the levels of minor bands were reduced (Figure 3B, right panels). The fractions containing recombinant APC were pooled for further analysis.

Purified recombinant APC was estimated to be ≥ 80% pure (Figure 3C). The yield of recombinant APC protein was estimated from the intensity of the Coomassie-blue-stained band for each recombinant APC relative to the intensity of a band representing 1 μg BSA (Figure 3C). The amount of recombinant APC purified from 2×10^9^ Sf9 cells was estimated to be 15, 250 and 200 μg for fl-APC, APC(1–1638) and APC(1–1311), respectively. The amount of fl-APC purified reflects the low expression levels relative to the expression levels of APC(1–1638) and APC(1–1311).

The identities of the purified recombinant APC proteins were confirmed using LC-MS/MS (Figure 3C). Each excised gel band contained peptides matching human APC (UniProt Accession number P25054). For fl-APC, 54 peptides derived from APC spanning residues 12–2581 (24% sequence coverage) were identified (Figure 3C) with a MASCOT protein score of 3179. For APC(1–1638), 42 peptides were identified spanning residues 12–1617 (40% sequence coverage, MASCOT protein score 1676) and 39 peptides spanning residues 25–1239 (51% sequence coverage, MASCOT protein score 2298) were identified for APC(1–1311). Other proteins identified (other than trypsin and keratin) had MASCOT scores of <70, indicating that levels of co-purifying proteins were not significant. The MS data confirmed that the purified recombinant proteins were the expected APC proteins.

## Discussion

The present study demonstrates the generation of APC mAbs that specifically recognise recombinant forms of APC, endogenous APC in solution and the location of APC in fixed cells. Our prediction that using a protein fragment encompassing the N-terminal oligomerisation domain of APC as the immunising antigen would generate antibodies that specifically recognise native, folded APC was correct: each of the APC-NT mAbs recognised native APC. The coiled-coil region was therefore a good antigen for raising monoclonal antibodies that can be used for isolation and detection of endogenous or recombinant, full-length or truncated APC. The APC-NT mAb clones recognized different epitopes and so we used a combination of the five APC-NT mAbs to develop an efficient affinity purification of recombinant APC.

Each of the APC-NT mAbs detected APC in characteristic clusters at the ends of microtubules in epithelial cells, which is consistent with the previously reported subcellular localisation of APC [24,25,27]. However, in contrast to a commercial APC antibody (H290), none of our APC-NT mAb clones stained the nucleus. There has been some controversy regarding the specificity of commonly used APC antibodies [28]. One APC antibody that was used to show nuclear localisation of endogenous APC was subsequently shown to cross-react with the DNA binding protein Ku80 [29]. Another report used biochemical fractionation and fluorescence microscopy to compare the localisation of APC as detected by a panel of the most commonly used APC antibodies, and found that most of the antibodies tested were unable to specifically detect APC in the nucleus [30]. In SW480 CRC cells that express truncated APC protein, the distribution was diffusely cytoplasmic, consistent with loss of the C-terminal microtubule binding regions of APC. Results from the five APC-NT mAbs reported here support the concept that endogenous APC is predominantly localised at the ends of microtubules in epithelial cells containing wild-type APC, a distribution that is lost upon APC truncation.

## Conclusions

In this study, the APC-NT mAbs were used for the purification of recombinant full-length and truncated APC. Recombinant APC that has been purified by this method can be used for further analysis of APC biochemistry. As these antibodies have been shown to recognise specifically endogenous, native APC, they will also be useful for the study of APC in cells and tissue samples, and for the isolation of APC-containing protein complexes in normal and cancer cells. These reagents will be useful for understanding the structure/function relationships of full-length and truncated APC and the interactions of APC with other cellular proteins.

## Methods

### Generation of APC monoclonal antibodies

APC monoclonal antibodies (APC-NT mAbs) were raised against a fragment of human APC (amino acids 1–61) (accession number P25054) with an N-terminal FLAG epitope tag, produced in *E.coli* (APC-NT). The fragment was amplified (Forward: 5′-CGCATATGTGCGATTACAAGGATGACGACGATAAGGGGGGGGCTATGGCTGCAGCTTCGTATGATC-3′, Reverse: 5′-GGCTGGATCCTTAAGCCATAGCTTCATC-3′) and cloned into pET30a + for *E.coli* expression. APC-NT was purified by affinity chromatography on anti-FLAG (M2) Sepharose beads (Sigma) followed by anion exchange (MonoQ HR10/10, GE Healthcare) and reversed-phase HPLC (Discovery C18 4.6 mm i.d. × 100 mm, Supelco).

Mice were immunised with 3× 30 μg purified APC-NT. Immunisation of mice, fusion of mouse spleens for production of hybridomas and initial screening for APC-NT mAbs was carried out by the WEHI Monoclonal Antibody Facility (Bundoora, Australia). Hybridomas were screened further for binding to the APC-NT antigen by ELISA and BIAcore analysis. Five clones (clones 2E7, 6D12, 6G6, 8D9, 9G11) were selected and purified by Protein-A chromatography. Antibodies were isotyped using Isostrip isotyping antibody kit (Roche).

### Immunoprecipitation of endogenous APC protein

MDCK cells [31] were grown in DMEM supplemented with 10% foetal calf serum and 1% penicillin/streptomycin. Colorectal carcinoma SW480 cells [22] were grown in RPMI supplemented with 0.01 μg/ml thioglycerol, 0.025 U/ml insulin, 1 μg/ml hydrocortisone, 10% FCS and 1% penicillin/streptomycin. Immunoprecipitations were performed as described [27]. Briefly, clarified cell lysates (1 mg) were incubated with APC-NT mAbs (2 μg) for 16 h at 4°C and precipitated with Protein G-Sepharose (100 μl, 10% slurry) (GE Healthcare) for 30 min at 4°C. Eluted proteins were analysed by SDS-PAGE (3–8% Tris Acetate NuPAGE gels) (Novex) and detected using anti-APC H290 antibody (Santa Cruz) followed by anti-rabbit IRDye800 antibody (LiCor) and imaged with the Odyssey infrared imaging system (LiCor).

### Biosensor analysis

All experiments were performed on BIAcore biosensor 2000 (BIAcore). APC-NT protein was exchanged into 10 mM NaAc pH 4.5 and immobilised on a CM5 chip using NHS-EDC as described previously [32]. Multi-determinant binding analysis was performed at a constant flow rate of 20 μl/min for 50 μl samples of each APC-NT antibody sequentially, as shown.

### Construction and expression of recombinant APC proteins

Fl-APC was cloned into the pBlueBacHIS2a in three fragments: Firstly, the 5′ end (1–617 bp) was PCR amplified to include a SacI site (Forward: 5′-GGTGGTGAGCTCATGGCTGCAGCTTCATATG-3′, Reverse: 5′-GGAAGAACAACTAGGTACCTGCCAGG-3′) and cloned into pGEM (Invitrogen). Secondly, the mid-region (618–6708 bp) was subcloned by digestion with KpnI and BstBI from pET30a-myc-APC [33]. Finally, the 3′ end (6709–8532 bp) was amplified by PCR to include a spacer, EE-tag [34] and SalI site (Forward: 5′-CTCCAACCAACAATCAGC-3′, Reverse: 5′-TACCTTGTGACATCTGTTCCCGGGGGGGAGTTCATGCCGATGGAGTGAGTCGACGGATCCATCC-3′) and cloned into pGEM. Each fragment was subcloned sequentially (SacI-KpnI, KpnI-BstBI, BstBI-SalI) into pBlueBacHIS2a.

Truncated APC(1–1638) and APC(1–1311) were cloned in two fragments. Firstly, the 5′ end (1–2156 bp) was amplified to include a 5′ SacI and a 3′ NcoI site (Forward: 5′-GGTGGTGAGCTCATGGCTGCAGCTTCATATG-3′, Reverse: 5′-GCACAAAATGATTGCC ATGGACCACC-3′) and cloned into pGEM. Separate 3′ ends were generated by PCR and cloned into pGEM for APC(1–1638) (2157–4914 bp) (Forward: 5′-GGTGGTCCATGGGAAGTGCTGCAGCTTTAAGGAATCTCAGGCAAATAGGCCTGCG-3′, Reverse: 5′-ACACCGGGGGATGATATGCCCGGGGGGAGTTCATGCCGATGGAGTGAG CGACAGTC-3′) and APC(1–1311) (2157–9327 bp) (Forward: 5′-GGTGGTCCATGGGAAGTGCTGCAGCTTTAAGGAATCTCAGGCAAATAGGCCTGCG-3′, Reverse: 5′-CCTCCTCAAACAGCTCAACCAAGCCCGGGGGGGAGTTCATGCCGATGGA GTGAGTCGACAGTC-3′). Each fragment was subcloned sequentially (SacI-NcoI, NcoI-SalI) into pBlueBacHIS2a.

pBlueBacHIS2a-APC cDNAs were co-transfected into Sf9 cells with linearised Bac-N-Blue™ DNA (Invitrogen). The resulting recombinant baculoviruses were plaque purified and amplified as described [35]. Sf9 (2×10^7^ cells) were infected with recombinant baculoviruses at a multiplicity of infection (MOI) between 1 and 5 pfu/ml to determine the optimum MOI for protein expression.

### Immunoprecipitation of recombinant proteins

Sf9 cells (2×10^7^ cells) were infected with the indicated baculoviruses at the optimised MOI. Cells were lysed in Triton X-100 (TX-100) Lysis buffer (Phosphate Buffered Saline (PBS) containing 1% TX-100 (Sigma) and protease inhibitors (Complete™ Protease Inhibitor Cocktail (Roche)) (500 μl). Lysates were clarified by centrifugation at 16000 × g for 10 min at 4°C and immunoprecipitated as described above.

### siRNA treatment and immunofluorescence

APC was depleted from MDCK cells using RNA interference as described [27]. MDCK and SW480 cells were lysed for immunoblot analysis or fixed for immunofluorescence as described [27]. Immunofluorescent staining was detected in successive focal planes using an Olympus FV1000 or Nikon C1 confocal microscope equipped with an Olympus 60× (NA 1.35) oil immersion lens or Nikon 60× NA1.35) water immersion lens using standard filter sets and laser lines.

### Affinity purification of recombinant APC

Sf9 cells (2×10^9^ cells) were infected with recombinant baculoviruses, harvested after 72 hrs, and lysed in TX-100 Lysis buffer + 20 mM imidazole. Recombinant APC proteins were affinity purified using IMAC on 1 ml Ni-NTA agarose (QIAgen) equilibrated in 20 mM imidazole, 2 mM β-mercaptoethanol in PBS, pH 7.5. Unbound proteins were removed with 20-column volumes of the same buffer. Bound APC was eluted with a step gradient of 5 ml 50 mM imidazole, 2.5 ml 100 mM imidazole and 2.5 ml 250 mM imidazole in the same buffer. Fractions were analysed by SDS-PAGE and Coomassie blue staining.

Recombinant APC was further purified by affinity chromatography using a mixture of the 5 APC-NT mAbs. Purified APC-NT mAbs (2E7. 6D12. 6D6, 8D9 and 9D11 5 mg total, 1 mg/clone) were covalently conjugated to Protein G-Sepharose (1 mg Ab/ml) using 50 mM Dimethyl Pimilimidate (Pierce) [36]. Coupling efficiency was determined by SDS-PAGE and Bradford assay, as previously described [36]. The APC-containing fractions from the Ni-NTA step were applied to APC-NT mAb Sepharose equilibrated with PBS containing 2 mM β-mercaptoethanol and 0.5 mM PMSF. Unbound proteins were removed with 20-column volumes of the same buffer. Bound APC proteins were eluted from the antibody column with two column volumes of 0.1 M Glycine, pH 3 containing 0.5 mM PMSF. Fractions (2 ml) were neutralised immediately with 1 M Tris, pH 8 and analysed by SDS-PAGE as above.

### Mass spectrometry

Coomassie blue-stained bands were excised from SDS-PAGE gels and digested in-gel with trypsin [37]. The resulting peptides were analysed by liquid chromatograph coupled to tandem mass spectrometry (LC-MS/MS) (LCQ-DECA, ThermoFinnigan) as described previously [37]. Peak lists were extracted as previously described [37] and searches performed against the LudwigNR protein sequence database (version Q209) (http://www.ludwig.edu.au/archive) using Mascot (version 2.2.04, Matrix Science) with settings of trypsin as the protease, carbamido-cysteine as fixed modification and oxidation of methionine and N-terminal acetylation as variable modifications. All assignments to APC were confirmed by manual inspection of the MS/MS spectra taking into account the Mascot ions score as well as the expectation value. Those peptide spectral matches falling below Mascot’s Identity or Homology threshold were accepted only if the annotated spectrum was in accordance with the known rules for peptide fragmentation (Eugene Kapp, personal communication).

## Abbreviations

APC: Adenomatous polyposis coli; MCR: Mutation cluster region; FAP: Familial adenomatous polyposis (FAP); IMAC: Immobilised metal affinity chromatography; MOI: Multiplicity of infection; mAb: Monoclonal antibody; DME: Dulbecco’s modified eagles media; MW: Molecular weight; siRNA: short interfering RNA; TX-100: Triton X-100; EE: Glu or EE epitope tag.

## Competing interests

The authors declare that they have no conflicts of interest with regard to this manuscript.

## Authors’ contributions

Conceived and designed the experiments: KLE BC MCF MJL AWB. Performed the experiments: KLE NLC JLC MCF. Analyzed the data: KLE MCF MJL. Wrote the paper: KLE AWB MJL MCF. All authors read and approved the final manuscript.
